# Oral-heart axis from pregnancy and postpartum: maternal oral microbiota relates with cardiac reverse remodeling

**DOI:** 10.1080/20002297.2026.2647506

**Published:** 2026-03-24

**Authors:** Juliana Morais, Maria João Azevedo, Ana Filipa Ferreira, Bernd W. Brandt, Egija Zaura, Mark J. Buijs, Adelino F. Leite-Moreira, Carla Ramalho, António Barros, Inês Falcão Pires, Benedita Sampaio-Maia

**Affiliations:** a RISE-Health, Departamento de Cirurgia e Fisiologia, Faculdade de Medicina, Universidade do Porto, Porto, Portugal; b Departamento de Ciências Funcionais, Escola Superior de Saúde, Instituto Politécnico do Porto, Porto, Portugal; c i3S - Instituto de Investigação e Inovação em Saúde, Universidade do Porto, Porto, Portugal; d Department of Preventive Dentistry, Academic Centre for Dentistry Amsterdam (ACTA), University of Amsterdam and Vrije Universiteit Amsterdam, Amsterdam, the Netherlands; e Cardiothoracic Surgery Department, Unidade Local de Saúde de São João, Porto, Portugal; f Departamento de Obstetrícia, Unidade Local de Saúde de São João, Porto, Portugal; g Departamento de Ginecologia-Obstetrícia e Pediatria, Faculdade de Medicina, Universidade do Porto, Porto, Portugal; h RISE-Health, Porto, Portugal; i Faculdade de Medicina Dentária, Universidade do Porto, Porto, Portugal

**Keywords:** Pregnancy, salivary microbiota, cardiovascular function, endothelial function, oral-heart axis

## Abstract

**Background:**

Emerging evidence links the oral microbiota to cardiovascular health, but this relationship remains poorly understood during pregnancy. Here, we investigated associations between the oral microbiota, cardiovascular physiology, and diet during and after pregnancy.

**Materials and methods:**

Salivary microbiota from 65 women in the 3rd trimester and 6 months postpartum were analyzed by 16S rRNA gene sequencing. Cardiovascular function was assessed via echocardiography, endothelial function by EndoPAT^TM^, nitric oxide levels by plasma nitrate/nitrite levels, and diet through the Food Frequency Questionnaire.

**Results:**

Left ventricular end-diastolic volume (LVEDV) was negatively associated with nitrite-reducing bacteria, namely, *Prevotella*, at both time points. Cluster analysis identified two reverse-remodeling profiles, one with poorer remodeling, greater postpartum weight retention and higher pregnancy microbial richness enriched with inflammation-associated genera that persisted postpartum. Higher *Porphyromonas* abundance during pregnancy predicted smaller postpartum LVEDV reductions. At postpartum, in the healthier cluster, *Neisseria* correlated with left ventricular mass changes and *Parvimonas* correlated with ΔLVEDV. Multivariate models confirmed independent microbiota–cardiac associations, while regression analyses did not support a clear diet–microbiota–cardiac axis.

**Conclusion:**

In conclusion, the salivary microbiota profile was associated with cardiovascular physiology during pregnancy and with postpartum cardiovascular recovery. These findings warrant confirmation in larger cohorts to clarify their clinical relevance.

## Key messages

This study suggests that mothers’ oral health during and after pregnancy is related to the cardiac reverse remodeling process:

- *The presence of Porphyromonas* during pregnancy predicts less favorable heart recovery after delivery.
- *After birth, higher levels of bacteria associated with oral health were linked to greater recovery of heart structure and function.*

## Graphical abstract

**Figure:**
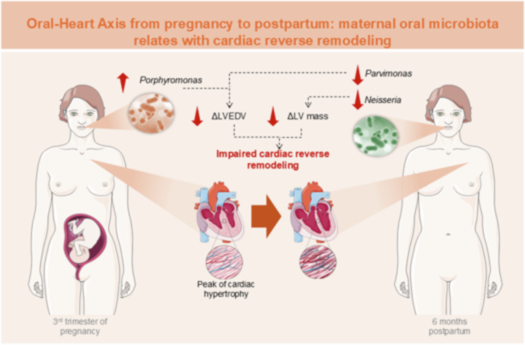

## Introduction

Over the course of gestation, profound physiologic changes occur in the woman's body, significantly affecting the endocrine, metabolic, immune, and microbial systems [1,2]. In the early period of pregnancy, an increase in systemic vasodilation and a higher cardiac output are observed [3]. These alterations persist throughout pregnancy, causing hypertrophy and dilation of the heart due to blood volume expansion, which normalises during the postpartum period through a process known as cardiac reverse remodelling [4].

Changes also occur in the oral cavity of pregnant women. An increase in hormones (oestrogen and progesterone) is associated with gingival inflammation and, consequently, periodontal diseases [5]. The maternal oral microbiota, which encompasses various oral niches [6], has gathered attention because of its potential impact on pregnancy outcomes. As reviewed by Saadaoui and colleagues (2021), a dysbiotic oral environment has been associated with miscarriage, preeclampsia, low birth weight, and preterm birth [7].

Despite physical distance, emerging evidence shows associations between oral dysbiosis and cardiovascular diseases [8]. Two possible mechanisms have been described to explain how the oral microbiota could contribute to systemic diseases. The first mechanism involves the direct entry of bacteria into the systemic circulation through an existing breach in the oral epithelium [9], which may be inflamed and ulcerated during pregnancy [10]. A meta-analysis of 63 studies concluded that 23 oral commensal bacterial species were present in atherosclerotic plaques, either individually or in co-existence [11]. Of these, 5 were unique to coronary plaques, while the remaining 18 were found in non-cardiac tissues [11]. The second mechanism involves the translocation of bacterial byproducts, toxins, and related pro-inflammatory cytokines that promote chronic low-grade inflammation in other tissues or affect cardiovascular physiology [12]. One of these products is the result of the microbial nitrate‒nitrite‒nitric oxide (NO) pathway. In saliva, nitrate-reducing bacteria convert nitrate to nitrite. In the stomach, owing to its acidic pH, nitrite is reduced to NO [13]. NO is a key molecule with vasoactive and endothelial protective effects associated with reduced blood pressure, ischaemia‒reperfusion injury and atherosclerosis [13].

In this study, we aimed to investigate the association between the salivary microbiota and cardiovascular health, specifically cardiac reverse remodelling and endothelial function, across the transition from late pregnancy to postpartum, and to assess whether maternal diet influences this oral-heart axis.

## Materials and methods

### Cohort design and ethical considerations

In this nested study within the longitudinal, prospective observational cohort PERIMYR/OralBioBorn, women were evaluated during the 3^rd^ trimester (3 T, 30‒35 gestational weeks) (*n* = 65) and 6 months postpartum (6MP, *n* = 63). Pregnant women were recruited at the Unidade Local de Saúde de São João (ULS São João), and the Unidade Local de Saúde de Matosinhos (ULSM). The exclusion criteria were age under 18 years, pre-existing cardiomyopathy, renal disease, chronic obstructive airway disease, active systemic infection, genetic syndromes, or type 1 diabetes *Mellitus*. The Ethical Committees of ULS São João and ULSM approved the study in 2018, with reference numbers 294/2018 and 86/CE/JAS, respectively. The study was conducted in accordance with the principles of the Declaration of Helsinki, and all eligible participants provided written informed consent to be included in this study.

### Clinical data collection

Detailed clinical data were collected through extensive questionnaires that included information about pregestational chronic disease, obstetric outcomes (gestational hypertension, gestational diabetes and weight gain), maternal smoking and oral healthcare habits.

### Maternal dietary habits assessment

A subgroup of 23 and 26 women completed the 86-item validated Food Frequency Questionnaire (FFQ) to assess their dietary intake during pregnancy and postpartum, respectively. The response options followed a 9-point frequency scale, ranging from ‘never’ to ‘≥6 times per day’. Data from the FFQ were used to explore the consumption of specific key food groups based on the Mediterranean Diet Adherence Screener [14]. The key food groups and respective intake thresholds studied included: red meat (≥2 servings/week); processed meat (≥5 servings/week); fish and seafood (≥3 servings/week); daily use of olive oil; daily use of butter; pastries (≥2 servings/week); pulses (≥2 servings/week); daily intake of nuts; carbonated/juice beverages (≥2 servings/week); fast food (≥1 servings/week); and daily intake of soup. These categories were classified as ‘YES’ or ‘NO’ based on whether the threshold was met.

### Echocardiography and endothelial function assessment

Echocardiographic assessment of cardiac function and structure was performed using a conventional transthoracic echocardiography performed using a 3 MHz phased-array probe (ACUSON SC2000 PRIME) by a single operator. Left ventricular (LV) mass (g) was estimated using the following formula: LV mass = 0.8 × {1.04 × [(LVIDd + PWTd + IVSd)³–(LVIDd)³]} + 0.6, where LVIDd = LV internal diameter at end-diastole, PWTdP = posterior wall thickness in diastole and IVSd = interventricular septal thickness in diastole. The biplane Simpson method was used to estimate the left ventricular end-diastolic volume (LVEDV), stroke volume and ejection fraction (EF). Additionally, E/e’ was calculated using the ratio of E mitral inflow velocity to the average early diastolic septal velocity with the lateral mitral annular velocity.

Endothelial function was assessed by measuring endothelium-mediated changes in vascular tone using two-finger probes on each index finger. This was performed with the non-invasive Peripheral Arterial Tone (PAT) signal technology system EndoPAT^TM^ 2000 (Itamar Medical, Israel). In brief, participants were placed in the supine position during the 15-minute assessment. Following a 5-minute rest period, a blood pressure cuff was inflated to one arm to a supra-systolic level (at least 60 mmHg above the systolic levels, typically between 200 and 300 mmHg) and maintained for 5 min to induce occlusion. After the cuff was released, reactive hyperaemia was monitored for an additional five minutes. The reactive hyperaemia index (RHI) was automatically calculated by the EndoPAT^TM^ software using the following formula: (C/D) ÷ (A/B), adjusted by a baseline correction factor. Endothelial dysfunction was considered when RHI was below 1.67 [15].

### Blood sample collection and indirect measurement of nitric oxide

Blood samples were collected and centrifuged for 15 min at 3857 × *g* at 4 °C. Nitric oxide production was estimated by measuring the relative levels of nitrate and nitrite in plasma (Invitrogen, Thermo Fisher). Briefly, to quantify nitrite concentrations, 25  μL of plasma was diluted 1:4 and analysed using the Griess reaction, a colorimetric assay. For nitrate determination, the remaining nitrate in the sample was enzymatically reduced to nitrite using nitrate reductase. The resulting total nitrite (the pre-existing nitrite plus nitrate that was converted) was then measured again with the Griess assay. The nitrate concentration was calculated by subtracting the initial nitrite value from the total nitrite value. The sum of both nitrite and nitrate concentrations represents the estimated total NO production.

### Clusters identification

To explore cardiac reverse remodelling profiles, we performed cluster analysis on echocardiographic changes (*Δ*) between 3T and 6MP, including ΔLV mass, ΔLVEDV, ΔEF and ΔE/e’. Among the 65 women included in the study, echocardiographic data at 6MP were available for 63, as two participants were lost to follow-up, and these missing values were imputed using the variable-specific mean. The optimal number of clusters was first estimated using the elbow method applied to the PCA scores. Based on this analysis, a two-cluster solution was selected. We then performed hierarchical clustering analysis on principal components (HCPC; *FactoMinerR* package, v. 2.11 [16]).

### Saliva collection

Participants were asked to collect unstimulated saliva 1 h after a meal or toothbrushing by passively drooling into a 50 mL sterile tube until 5 mL was collected. All samples were placed on ice until they were divided into 1 mL aliquots, after which they were stored at −80 °C until further analysis.

### Microbial analysis and data processing

Bacterial DNA extraction and quantification were described previously [17]. In brief, DNA was isolated from 200  μL of each saliva samples using the MagMini DNA isolation kit (LGC Genomics Ltd., UK). Quantification of the extracted DNA was carried out via qPCR using universal primers targeting the 16S rRNA gene. Sequencing was conducted on the MiSeq system (Illumina, San Diego, CA, USA) at TNO (Zeist, The Netherlands). The paired-end reads obtained from the MiSeq platform were merged and quality-filtered, and contaminant PhiX sequences were removed. High-quality reads were denoised using the UNOISE algorithm (USEARCH v10.0.240), producing zero-radius operational taxonomic units (zOTUs). Representative zOTU sequences were taxonomically assigned using the Human Oral Microbiome Database [18] as previously described [19]. Microbiome data analysis was performed using R (version 4.4.3 [20]). Based on the distribution of reads across samples and to retain sufficient depth while minimising sample loss, the samples were randomly subsampled to an equal depth of 4903 using the *rarefy_even_depth* function (*phyloseq* v. 1.50.0. [21]). Alpha diversity was calculated on the rarefied dataset (4903 counts per sample) at the zOTU level using the *estimate_richness* function from the *phyloseq* package. For beta diversity and taxonomic composition analyses, counts were normalised to relative abundances using Total Sum Scaling (TSS). No Centred Log Ratio (CLR) transformation was applied, as recent evidence suggests that CLR may retain sequencing depth-related signals that can affect ordination-based analyses [22]. Instead, samples were rarefied to a common sequencing depth to control for uneven sequencing effort.

### Statistical analysis

Clinical descriptive statistics were performed by SPSS version 30 (IBM SPSS Statistics, Chicago, IL, USA). Continuous variables are expressed as medians with interquartile ranges, and categorical variables are expressed as absolute values and relative frequencies. Clinical and microbial differences between clusters at each timepoint were evaluated using non-parametric tests (Wilcoxon rank-sum test for continuous variables and Fisher's exact test for categorical variables). Statistical significance was considered if *p* < 0.05.

To explore associations between the salivary microbiota composition and echocardiographic/vascular variables, biplots of relative abundances at the genus level were generated for each timepoint. The environmental fitting of variables onto the PCA ordination was conducted with the *envfit* function (999 permutations, vegan R package v. 2.6.10. [23]). Genera showing strong associations in biplots (based on the envfit results) were subsequently included in linear regression analyses to quantify their associations with selected echocardiographic parameters. Multivariable linear regression models were fitted with echocardiographic measurements as dependent variables, adjusting for clinical covariates previously identified as key determinants of cardiac structure and function [24], namely, previous hypertension, pregestational obesity, smoking habits and weight change (weight gain up to 3T or weight retention at 6MP, depending on the analysis timepoint). This model is referred to as the ‘clinical model’ hereafter.

Alpha-diversity indices, namely, the Shannon, Chao1, and observed richness, were estimated on a rarefied dataset using the *estimate_richness* function (*phyloseq* v. 1.50.0 [21]). and compared between cardiac reverse remodelling clusters using the Wilcoxon rank-sum test. For alpha-diversity indices showing significant differences between clusters, sensitivity analyses were performed using linear regression models with cluster membership as the main predictor, adjusting for maternal age and pre-pregnancy body mass index (BMI).

For each timepoint, the beta diversity was assessed using Bray–Curtis distances computed from zOTU-level relative abundance data (*distance*, method = ‘bray’ from the *phyloseq* package), and principal coordinate analysis (PCoA) was performed with the *ordination* function. Differences in microbial community composition between clusters were tested by permutational multivariate analysis of variance (PERMANOVA) with 9999 permutations, as implemented in the *adonis2* function (*vegan* package), after verifying the homogeneity of multivariate dispersions (*betadisper*).

Comparisons of genus-level relative abundance between clusters were performed using Wilcoxon rank-sum tests with the Benjamini‒Hochberg false discovery rate (FDR) correction method. For visualisation purposes, volcano plots were generated to illustrate the magnitude of differences (Cluster 2–Cluster 1, based on mean relative abundance) against the strength of the association (−log10 *p*-value). In these plots, unadjusted *p*-values were used to better display overall trends, whereas formal statistical interpretation relies exclusively on FDR-adjusted values. To explore potential relationships between bacterial genera and clinical variables, Spearman correlation coefficients were computed using *corr.test* function (*psych* package, v. 2.5.6. [25]). Correlation matrices were visualised as clustered heatmaps with the *pheatmap* package v 1.0.13. [26]). Associations between genus-level taxa that were significantly different between clusters and dietary variables were explored using Spearman’s correlations. Further regression analysis was performed to examine the interplay between diet, oral microbiota, and echocardiographic parameters. During data analysis, artificial intelligence-based tools (ChatGPT, OpenAI and GPT-5.2) were used to assist in draughting and debugging R scripts. All analyses were reviewed, validated and interpreted by the authors.

## Results

### Participants characterisation

A total of 65 pregnant women were recruited and followed from April 2019 to January 2022. The participating women had a median age of 35 years [IQR: 32; 37] at the moment of the first visit (3T) and a median pregestational BMI of 23.1 [IQR: 21.5; 28.3] kg.m^−2^. Most of the participants (67%) were highly educated and held a bachelor's degree (41.5%), master’s degree (20%) or doctoral degree (6%), and 7.7% smoked during the 3T of pregnancy. Maternal sociodemographic and clinical data are reported in Table 1.

**Table 1.** Clinical characteristics of the participants in the study.

Maternal clinical data (*n* = 65)	
Maternal age (years), median [IQR]	35 [32; 37]
Education level, *n* (%)	
Basic	1 (1.5)
High school	20 (30.8)
Bachelor’s degree	27 (41.5)
Master’s degree	13 (20.0)
Doctoral degree	4 (6.2)
Smoking habits, *n* (%)	
Yes	5 (7.7)
No	60 (92.3)
Pregestational BMI (kg.m^−2^), median [IQR]	23.1 [21.5; 28.3]
Pregestational BMI category, *n*(%)	
Underweight	1 (1.5)
Normal weight	39 (60.0)
Overweight	14 (21.5)
Obesity	11 (17)
Pregestational diabetes (T2D), *n* (%)	1 (1.5)
Gestational diabetes, *n* (%)	8 (12.5)
Chronic hypertension, *n* (%)	14 (21.5)
Gestational hypertension, *n* (%)	1 (1.5)
Gestational weight gain (kg), median [IQR]	10.1 [7.00; 13.0]
Postpartum weight recovery at 6MP (kg), median [IQR]	2.14 [-1.00; 6.00]
Gestational age at birth (weeks), median [IQR]	39 [38; 39]

Continuous variables are expressed by median [interquartile range]. BMI, body mass index; IQR, interquartile range; T2D, Type 2 diabetes.

Regarding maternal oral care, at 3 T of pregnancy, most women brushed their teeth 2 times a day (61.5%) and used a toothbrush complement, such as an elixir, dental tape, or water jet (83.1%) (Table S1). In the year before the first visit (3T), 21.5% of participants never went to the dentist, 32.3% and 20.0% went once or twice, respectively, primarily for dental cleaning (Table S1).

Among the participants who completed the study and provided saliva samples at both visits (3T and 6MP), only 23 filled the FFQ at the 3rd trimester and 28 – at 6 months postpartum. With these data, no differences were found in the consumption of the food items explored (Table S2).

Regarding the cardiovascular characteristics, we observed significant differences between the gestational period and the postpartum period (Table S3). Echocardiographic assessment revealed a significant reduction in relative wall thickness (0.35 [0.31; 0.40] vs 0.31 [0.28; 0.36], *p* < 0.001), LV mass (32.9 [28.0; 38.2] vs 26.7 [23.0; 30.2], *p* < 0.001), and LVEDV (24.5 [22.7; 26.8] vs 21.8 [19.7; 23.8], *p* < 0.001). While the stroke volume and ejection fraction remained unchanged, the E/E’ ratio decreased (6.76 [5.76; 7.89] vs 5.70 [5.22; 6.51], *p* = 0.001) (Table S3).

The endothelial function was assessed through the RHI, which did not significantly change (1.53 [1.39; 1.73] vs 1.50 [1.32; 2.18], *p* = 0.676) (Table S3). After categorising participants in two groups regarding the RHI cut-offcutoff of 1.67, an indicator of endothelial dysfunction, the prevalence of endothelial dysfunction decreased from 71.7% at 3T to 60.0% at 6MP. However, this difference did not reach statistical significance (*p* = 0.254). Despite no differences observed in nitrate levels between the two timepoints, there was a significant reduction in nitrate-derived estimates of NO production (454.1 [383.9; 658.5] vs 330.8 [256.0; 460.4], *p* = 0.007) and nitrite levels (383.99 [331.6; 466.8] vs 285.4 [236.7; 369.8, *p* < 0.0019 (Table S3).

### Association between oral microbiota and cardiovascular structure and function

**Figure 1.**
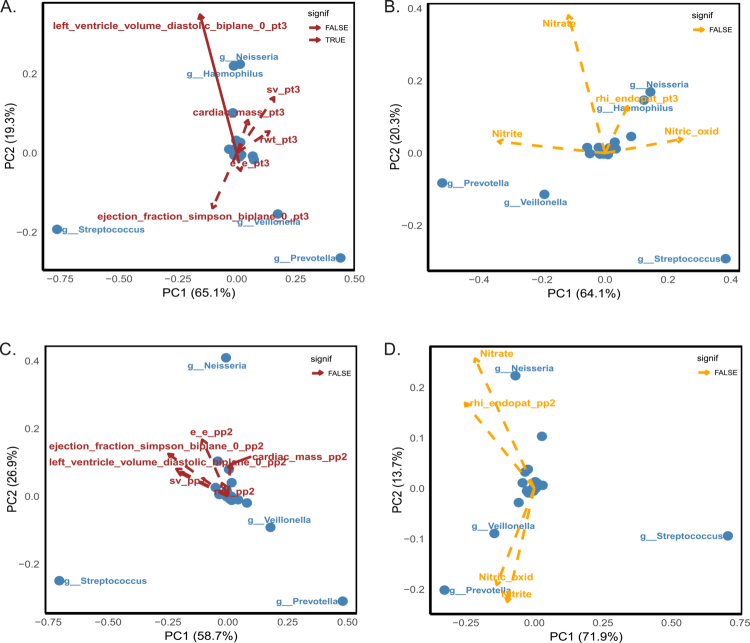
Principal component analysis (PCA) biplots illustrating the relationship between bacterial genera and cardiovascular outcomes during pregnancy (A and B) and six months postpartum (C and D). Panels A and C depict associations with echocardiographic parameters (red vectors), whereas panels B and D show endothelial function and nitric oxide-related biomarkers (yellow vectors). The blue dots represent individual bacterial genera. The direction and length of the vectors reflect the direction and strength of the correlations. The solid arrows indicate statistically significant associations (*p* < 0.05), while dashed arrows denote non-significant trends.

To explore the relationship between the oral microbiota and cardiovascular parameters, we generated biplots illustrating the distribution of bacterial genera in relation to echocardiographic and endothelial parameters at 3T and 6MP (Figure 1). During pregnancy, *Prevotella* and *Veillonella* were positioned in the opposite direction as the vector for LVEDV (left_ventricle_volume_diastolic_biplane_pt3) (Figure 1A). On the other hand, *Haemophilus* and *Neisseria* were also located in the same direction as the LVEDV and closer to the vector of stroke volume (sv_pt3) (Figure 1A), RHI (rhi_endopat_pt3), and plasma nitrate (nitrate) and indirectly measured NO (Nitric_oxid) (Figure 1B). In the postpartum period, despite *Prevotella* and *Veillonella* remaining positioned in the quadrant opposite to the LVEDV vector, the cardiac parameters are short and centrally located, indicating a limited contribution to the variance explained by PC1–PC2, whereas the dispersion of oral genera appears to account for most of the visible separation among participants (Figure 1C). With respect to the vascular markers, *Neisseria* was associated with the direction of the RHI and nitrate vectors, suggesting a positive association, although this pattern did not reach statistical significance (Figure 1D).

Based on the PCA results, in which only the LVEDV showed meaningful correlations with bacterial genera (Figure 1A), this variable was selected for further statistical modelling. Linear regression models were constructed to explore associations between LVEDV and the five genera that were most prominent in the PCA biplots (*Neisseria*, *Haemophilus*, *Veillonella*, *Prevotella,* and *Streptococcus*) (Table S4). During pregnancy, in both the unadjusted model (*β* = –34.3, *p* = 0.026) and the clinical model adjusted for arterial hypertension, obesity, smoking habits, and weight gain until the 3 T visit (see methods section), the relative abundance of *Prevotella* (*β* = −40.3, *p* = 0.004) remained significantly associated with LVEDV (Table S4). At 6MP, *Prevotella* remained significantly associated with the LVEDV only in the adjusted clinical model (*β* = −26.8, *p* = 0.025) (Table S4). No significant associations were observed for *Haemophilus*, *Veillonella*, or *Streptococcus* in either model (Table S4).

### Cluster of participants based on cardiac reverse remodelling profile

**Figure 2.**
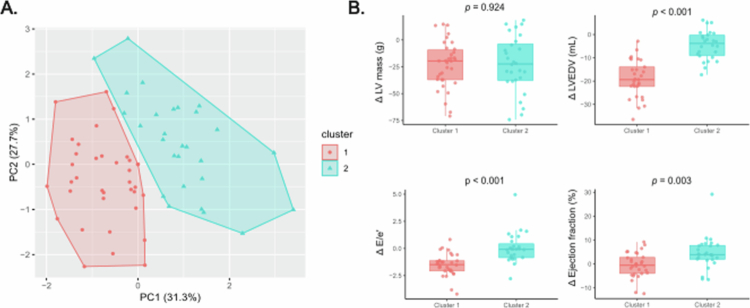
Clustering of participants according to dynamic changes (*Δ*) in echocardiographic parameters between 3T and 6MP. (A) Principal component analysis (PCA) plot showing the projection of the HCPC-defined clusters, with Cluster 1 (red) and Cluster 2 (blue). (B) Boxplots of the echocardiographic *Δ*-variables used to define the clusters, including the ΔLV mass, ΔLVEDV, ΔE/e’ and *Δ* Ejection fraction.

Given the variability observed in echocardiographic parameters at 6MP, we stratified participants into clusters reflecting different cardiac reverse remodelling trajectories, with 2 identified clusters (Figure 2A). Cluster 1 (*n* = 37) comprised women with a more favourable cardiac reverse remodelling profile, showing greater recovery in three of the assessed parameters, namely, the LVEDV, E/e’, and ejection fraction (Figure 2B). In contrast, Cluster 2 (*n* = 28) had incomplete or less efficient postpartum reverse remodelling, characterised by significantly smaller changes in LVEDV, E/e’, and ejection fraction, despite a similar reduction in LV mass compared with Cluster 1 (Figure 2B).

When comparing maternal characteristics between clusters, women in Cluster 2 had significantly greater weight retention during the first 6 months postpartum than those in Cluster 1 (Table S5). No significant differences were observed for age, pre-existing obesity, diabetes, hypertension, gestational complications, and gestational weight gain up to T3 (Table S5). Subsequent microbiota analyses were conducted according to these clusters to explore potential microbial signatures of divergent recovery patterns.

### Alpha- and beta-diversity of the oral microbiota in the cardiac reverse remodelling clusters

**Figure 3.**
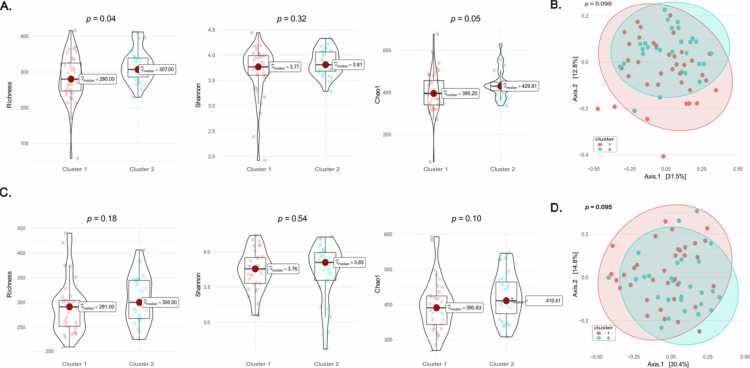
Comparison of oral microbiota diversity indices between the two clusters of cardiac reverse remodelling at 3T (A and B) and at 6MP (C and D). Alpha-diversity indices (richness, Shannon diversity index, and Chao1) are shown as violin plots (A and C). Beta diversity was measured by Bray‒Curtis distances using principal coordinate analysis (PCoA) (B and D).

At 3T, Cluster 2 showed significantly higher community richness (*p* = 0.04, Figure 3A). However, in sensitivity analyses adjusting for maternal age and pre-pregnancy BMI, this difference was attenuated and did not remain statistically significant (adjusted *p* = 0.074). Other alpha-diversity indices, including the Shannon and Chao1 diversity did not differ between the clusters (Figure 3A). Similarly, beta-diversity analysis based on Bray‒Curtis distances revealed no separation between clusters at 3T (PERMANOVA *p* = 0.099, Figure 3B), and no differences were observed in any alpha-diversity index (Figure 3C), nor in beta diversity among clusters (PERMANOVA *p* = 0.095, Figure 3D).

#### Taxonomical changes of the oral microbiota in the cardiac reverse remodelling clusters and network with cardiovascular outcomes

The overall taxonomic distribution was broadly similar between the two clusters and across time. At the phylum level, Bacillota dominated in both groups during pregnancy (~54–56%), followed by Bacteroidota (17%) and Pseudomonadota (12–14%) (Table S6). At 6MP, the relative abundance of the Absconditabacteria phyla was significantly higher (0.016% vs 0.049%, *p-adj* = 0.04), as well as Fusobacteriota with a significant nominal difference (3.10% vs 3.87%, *p* = 0.030) (Table S6). At the genus level, *Streptococcus* was consistently the most prevalent genus during pregnancy (~39–40%), followed by *Prevotella* (13–14%) and *Veillonella* (8–10%) (Table S7). After delivery, we observed an increase in the relative abundance of *Porphyromonas* (1.203% vs 2.293%, *p* = 0.001) and *SR1_G-1* (0.016% vs 0.049%, *p* = 0.04) (Table S7).

**Figure 4.**
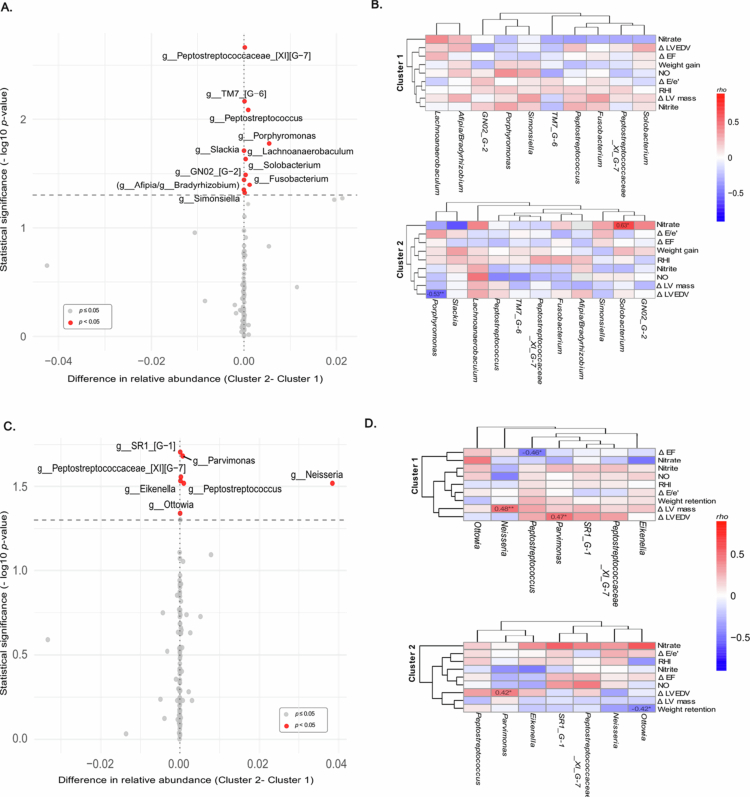
Taxonomix changes between clusters at 3T (A and B) and at 6MP (C and D) and association with clinical variables. Volcano plots showing genus-level differences in relative abundance between the two cardiac remodelling clusters at 3T (A) and at 6M (C). Heatmaps of Spearman correlations between the differentially abundant bacterial genera and echocardiographic and clinical variables at 3T (B) and at 6M (D). The colours represent correlation coefficients (red = positive, blue = negative), with significance indicated in the boxes (*p < 0.05).

Although between-group comparisons identified several nominal differences in the relative abundance, many of these genera were present at an extremely low abundance and should therefore be interpreted with caution. During 3T, women in Cluster 2 exhibited a higher relative abundance of *Peptostreptococcaceae_[XI][G-7]* (0.023% vs 0.005%, *p* = 0.002), *TM7_[G-6]* (0.023% vs 0.011%, *p* = 0.007), *Peptostreptococcus* (0.244% vs 0.152%, *p* = 0.008), *Porphyromonas* (1.512% vs 0.969%, *p* = 0.017), *Slackia* (0.000% vs 0.001%, *p* = 0.019), *Lachnoanaerobaculum* (0.220% vs 0.185, *p* = 0.023), *Solobacterium* (0.087% vs 0.052%, *p* = 0.032), *GN02_[G-2]* (0.000% vs 0.002%, *p* = 0.036), *Fusobacterium* (1.411% vs 1.290%, *p* = 0.040) and *Simonsiella* (0.016% vs 0.002%, *p* = 0.045) compared to Cluster 1 (Figure 4A; Table S8). Cluster 1 showed higher relative abundance of taxa assigned to the genera *Afipia* or *Bradyrhizobium* (which could not be distinguished via 16S rRNA analysis) (0.003 vs 0.001, *p* = 0.044) (Figure 4A; Table S8).

Genera that showed significant differences in relative abundance between clusters were further explored for correlation with *Δ*-echocardiographic parameters, endothelial biomarkers, and gestational weight gain or postpartum weight retention (Figure 4B). No associations were observed between the levels of the mentioned genera and clinical variables in Cluster 1. On the other side, in Cluster 2, we observed a strong positive correlation between *Solobacterium* and nitrate levels (rho = 0.63, *p* = 0.036), as well as a negative association between *Porphyromonas* and ΔLVEDV (rho = −0.53, *p* = 0.005). After adjustment for the clinical model (adjusted for arterial hypertension, obesity, smoking habits and weight gain until 3T), the correlation observed between *Solobacterium* and nitrate levels was not confirmed (ẞ = 20.4, *p* = 0.728), indicating that this association was not robust. In contrast, *Porphyromonas* remained independently associated with a lower ΔLVEDV (ẞ = −280.1, *p* = 0.003). The adjusted model explained 38% of the variance in ΔLVEDV (adjusted R^2^ = 0.38).

At 6MP, Cluster 2 also showed nominal significantly higher proportion of *SR1* (0.054% vs 0.045%, *p* = 0.020), *Parvimonas* (0.155% vs 0.083%, *p* = 0.021), *Peptostreptococcaceae_[XI][G-7]* (0.034% vs 0.009%, *p* = 0.028)*, Eikenella* (0.028% vs 0.014%, *p* = 0.029), *Neisseria* (10.6% vs 6.73%, *p* = 0.030), *Peptostreptococcus* (0.271% vs 0.172%, *p* = 0.030) and *Ottowia* (0.011% vs 0.004%, *p* = 0.046) (Figure 4C; Table S9). Among participants of Cluster 1, higher levels of *Neisseria* and *Parvimonas* were significantly correlated with ΔLVmass (rho = 0.48, *p* = 0.006) and ΔLVEDV (rho = 0.47, *p* = 0.013), respectively. After adjustment for the clinical model (adjusted for arterial hypertension, obesity, smoking habits and weight retention until 6MP visit), both genera remained associated with the ΔLV mass (*Neisseria*: ẞ = 181.5, *p* = 0.023 and *Parvimonas*: ẞ = 6537.2, *p* = 0.046). A negative correlation was observed between *Peptostreptococcus* and ΔEF (rho = -0.46, *p* = 0.017) and confirmed by the clinical model (ẞ = -999.6, *p* = 0.013) (Figure 4D).

As observed in Cluster 1, in Cluster 2, it was also observed that *Parviromonas* correlated with ΔLVEDV (rho = 0.42, *p* = 0.033), but this association was lost after adjusting for the clinical model (ẞ = 1121.8, *p* = 0.203). In addition, in Cluster 2, a negative correlation was observed between *Ottowia* and weight retention (rho = −0.42, *p* = 0.026).

### Interaction between diet-oral microbiota and cardiovascular parameters

**Figure 5.**
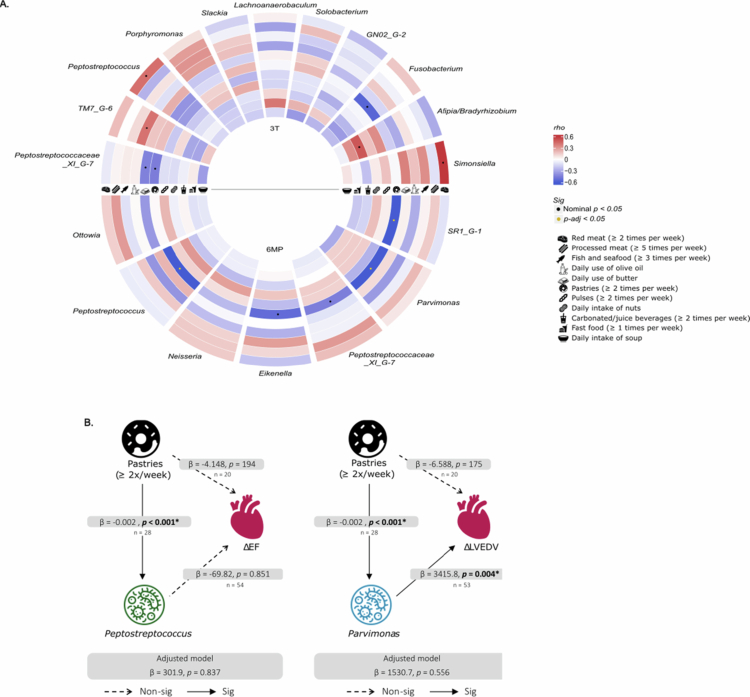
Associations between diet and the oral microbiota. (A) Heatmap showing Spearman correlations between bacterial genera that showed nominal differences and maternal intake of selected food groups during pregnancy and postpartum. (B) Regression models exploring the relationships among ΔEF or ΔLVEDV (dependent variables) and Peptostreptococcus or Parvimonas, respectively, adjusted for pastry consumption.

We further explored whether bacterial genera showing nominal differences between clusters were associated with dietary habits (Figure 5). In the third trimester, several nominal correlations were found between diet and bacterial genera. *Simonsiella* was positively correlated with red meat intake (≥2 times per week; rho = 0.50, *p* = 0.016). higher consumption of pastries during the 6MP was negatively associated with several genera after FDR correction, including adjusted *p*-values, namely, *SR1_G-1* (rho = −0.72, *p-adj* = 0.002), *Parvimonas* (rho = −0.69, *p-adj* = 0.003) and *Peptostreptococcus* (rho = −0.61, *p-adj* = 0.016) (Figure 5A). To evaluate whether pastry intake accounted for the association between *Peptostreptococcus* and ΔEF, and between *Parvimonas* and ΔLVEDV, we fitted regression models adjusted for pastry consumption. After adjustment, neither association remained statistically significant, indicating that these relationships were not independent of pastry intake (Figure 5B).

## Discussion

In this study, we characterised maternal cardiovascular remodelling from late pregnancy to 6 months postpartum and investigated its associations with the salivary microbial composition and dietary habits as part of a larger project in which the cardiovascular [24] and oral microbiota [19] components were independently explored. As expected, we observed a postpartum normalisation of cardiac structure and diastolic function to pre-gestational values, including reductions in relative wall thickness, LV mass, LVEDV and E/e’. Interestingly, our initial analysis revealed that LVEDV was negatively associated with nitrate-reducing oral bacteria, particularly *Prevotella*, at both time points. We then identified two distinct cardiovascular remodelling profiles through cluster analysis. One subgroup of women exhibited less efficient postpartum cardiac recovery and greater weight retention, and was characterised by higher microbial richness in unadjusted analyses during pregnancy and a sustained abundance of inflammation-associated genera such as *Porphyromonas*. In contrast, health-associated taxa, including *Neisseria* and *Parvimonas* are associated with more favourable remodelling trajectories.

There is limited published data available that links cardiac structure and function to changes in the oral microbiota [8]. In our cohort, we observed that the relative abundance of *Prevotella,* a Gram-negative bacterial genus, was inversely associated with the LVEDV at 3T and at 6MP even after adjustment for the clinical model. LVEDV is defined as the telediastolic volume and is a structural marker of cardiac remodelling. While in pregnancy, a higher LVEDV typically reflects a better capacity of the left ventricle to accommodate increased blood volume, in the postpartum period, the LVEDV is expected to decrease to pregestational volumes [27]. Previous studies have shown associations between a higher proportion of bacteria from the genus *Prevotella* and hypertension [28] and the risk of cardiovascular disease [29], by triggering pro-inflammatory mechanisms. Specifically, during pregnancy, increased progesterone is associated with an increase in *Prevotella* spp., many of which are associated with periodontal diseases [30]. In addition, *Prevotella* is listed as one of the nitrate-reducing bacteria capable of influencing the NO production and thereby enhancing vasodilation [31].

Beyond their ability to activate inflammatory pathways and induce NO production, several *Prevotella* strains metabolise sulphur-containing amino acids, producing hydrogen sulphide (H_2_S) [32,33]. H_2_S has been shown to increase NO bioavailability through synergetic signalling interactions [34]. As NO is a potent endothelial vasodilator, higher NO levels reduce systemic vascular resistance and, consequently, lower afterload, which may contribute to a smaller LVEDV. Given this, we hypothesise that lower *Prevotella* abundance results in lower NO production (directly and indirectly via H_2_S), leading to less vasodilation and, therefore, higher LVDEV.

In line with the physiological cardiovascular adaptations known to occur during and after pregnancy [24,35], the echocardiographic findings of this study demonstrate a pattern of postpartum cardiac remodelling and functional recovery, as evidenced by significant reductions in relative wall thickness, LV mass, LVEDV and E/e’ at 6MP. These changes suggest a reversal of volume and pressure overload, typically in late gestation, corresponding to the normalisation of hemodynamic demands postpartum. However, we found two different clusters regarding the cardiac reverse remodelling profile, with Cluster 2 showing less favourable cardiac reverse remodelling.

During pregnancy, Cluster 2 showed a tendency toward higher community richness with increased levels of inflammatory bacterial genera, namely, *Peptostreptococcus [36]*, *Porphyromonas [37]*, *Fusobacterium [38]* and *Solobacterium* [39]. Among these genera*, Porphyromonas* remained associated with a lower ΔLVEDV after adjustment for the clinical model, suggesting its potential role as a predictor of poorer cardiac reverse remodelling. Although an additional 20 species exist within this genus, the available literature has focused mainly on the systemic effect of *Porphyromonas gingivalis [37]*. This bacterium is highly prevalent in the human oral cavity and possesses several virulence features, including the presence of gingipains and lipopolysaccharides (LPS) in its outer membrane, which can activate the Toll-like receptors TLR2 and TLR4 and thereby trigger inflammation [40]. As reviewed by Zhang et al., the inflammatory state induced by *Porphyromonas* spp. contributes to endothelial oxidative stress, upregulating inducible nitric oxide synthase (iNOS) and downregulating endothelial nitric oxide synthase (eNOS) [40]. Thus, a reduction in NO-mediated vasodilation leads to increased afterload, which may contribute to increased LVEDV, as observed in our cohort. Supporting this mechanism, a recent study in mice treated with *P. gingivalis* reported elevated systemic pro-inflammatory markers, a reduced ejection fraction, increased LV dimensions on echocardiography, and increased myocardial levels of matrix metalloproteinases [41].

During the postpartum period, Cluster 2 no longer presented increased alpha diversity but continued to show increased abundances of *Peptostreptococcaceae_[XI][G-7]* and *Peptostreptococcus* compared to Cluster 1, as well as increased levels of *Parvimonas*, *Eikenella*, *Neisseria* and *Ottowia*. In our study, we showed a positive correlation between *Parvimonas* and the change in LVEDV in both clusters, suggesting that a higher abundance of this genus may be linked to more favourable reverse remodelling. However, in Cluster 2, this association was lost after adjustment for the clinical model. The genus *Parvimonas* has only three species identified to date (https://lpsn.dsmz.de/search?word=parvimonas), with *P. micra* being the most common [42]. *P. micra* is not only associated with oral infections but also with endocarditis [43] because of its high proteolytic activity and H_2_S toxicity, as reviewed by Shimizu [44]. More studies are needed to confirm the association between *Parvimonas* and greater LVEDV.

In our study, we observed that the *Ottowia* genus was associated with greater postpartum weight retention in Cluster 2, which showed higher weight retention compared with Cluster 1. Although *Ottowia* is not a highly abundant or frequently reported genus in oral microbiota studies, it is significantly enriched in the oral microbiota of healthy individuals when compared with the myocardial infarction group [45].

Cluster 1, characterised by a more favourable cardiac reverse remodelling profile, showed a higher abundance of *Neisseria* than Cluster 2. Interestingly, within this cluster, higher levels of *Neisseria* were positively associated with greater recovery of the LV mass during the postpartum period. Certain species of *Neisseria* have been linked to endothelial activation [46], which may contribute to subtle cardiovascular adaptations. Growing evidence supports the role of the oral microbiota in modulating systemic vascular tone via the NO pathway. As *Neisseria* is a well-recognised nitrate-reducing bacterium [47], enhanced activity of the enterosalivary nitrate‒nitrite‒NO pathway and thereby the bioavailability of NO. This could be one plausible mechanism that relates higher levels of *Neisseria* to improved LV mass recovery. It was also observed that higher levels of *Peptostreptococcus* were also associated with a smaller change in the ejection fraction, suggesting that the presence of this bacterial genus may impair cardiac reserve remodelling. *Peptostreptococcus* genus is a Gram-positive anaerobic coccus that can act as a commensal organism but also as an opportunistic pathogen, which is typically linked to oral infection and, although rare, with infective endocarditis [48]. However, there is evidence that *Peptostreptococcus* spp. can induce an inflammatory state capable of affecting cardiac function [49], no direct mechanism has yet been identified to explain its influence on the ejection fraction.

Noteworthy findings confirm the impact of pastries on the oral microbiota, showing that this effect is evident in pregnant women during pregnancy and becomes particularly significant after birth. In general, we found that participants who frequently consumed pastries during pregnancy had lower levels of *Peptostreptococcaceae_[XI][G-7]* and *Fusobacterium*. After birth, pastries promoted lower levels of *Peptostreptococcaceae_[XI][G-7], Parvimonas*, *Peptostreptococcus*, *Eikenella* and *SR1_G-1*. These findings seem counterintuitive because they suggest that the consumption of sugar and refined carbohydrates could have a potential protective effect against certain pathogenic bacteria, as these genera are often associated with inflammation [50]. The shift in bacterial populations might not necessarily confer a health benefit but rather indicate a microbiome adjustment to dietary patterns that are generally considered less healthy, such as frequent pastry consumption. A previous study that included 8,173 adolescents showed that high salivary glucose, which is associated with the intake of pastries, reduced the overall bacterial load [51]. The authors propose a possible hypothesis for this phenomenon: the increase in salivary glucose caused by hyperglycaemia status promotes higher levels of bacteria that synthesise acidic metabolites that, owing to the lower levels of pH in the oral cavity, promote a decrease in salivary bacterial numbers with a change in bacterial composition [51].

Despite the valuable and fresh insights provided by this study on the oral-cardiovascular axis, several limitations must be acknowledged. First, the sample size was small, as was the number of saliva and blood samples collected at each timepoint, which has limited the robustness of the analyses. This constraint is likely related to the fact that the cohort was assembled during the COVID-19 pandemic. As a result, the FFQ was administered exclusively through an online form, which significantly reduced the response rate and limited the possibility of conducting paired analyses, since not all participants responded at both timepoints. In addition, owing to pandemic-related restrictions, oral clinical examinations could not be performed in all participants, with full oral assessments obtained in only 17 of the 65 participants. Additionally, saliva samples were not collected under standardised conditions (e.g. at the same time of day), thereby not respecting the circadian variations in the oral microbiota [52]. Another limitation is the method we used to sequence the oral microbiota, which does not allow for conclusions regarding species-level or microbial functions. Finally, while NO is a highly reactive and short-lived molecule, its quantification through its stable precursors (nitrite and nitrate) provides an indirect, though generally reliable, estimation of total NO production.

## Conclusions

This longitudinal study highlights a bidirectional relationship between the oral microbiota and maternal cardiovascular physiology during the transition from late pregnancy to the postpartum period. Notably, women exhibiting less favourable cardiac reverse remodelling postpartum, characterised by smaller reductions in LVEDV and greater weight retention, had a higher tendency toward microbial richness during pregnancy, along with a persistent overrepresentation of inflammation-associated genera such as *Porphyromonas*. This pattern suggests that a pro-inflammatory oral microbiota may be linked to delayed cardiovascular recovery. In contrast, genera such as *Neisseria*, which is known for nitrate-reducing activity and potential vascular benefits, and *Parvimonas*, which emerged in association with greater improvements in cardiac parameters, were more abundant in women with healthier cardiovascular trajectories. While no direct causal link between diet, microbiota, and cardiac remodelling could be established, our findings point to specific microbial signatures that may reflect cardiovascular adaptation across this critical period. These insights support the concept of an oral-heart axis in maternal health, open promising avenues for novel, non-pharmacological strategies to enhance cardiovascular recovery, and underscore the need for further studies to elucidate underlying mechanisms and confirm clinical relevance.

## Supplementary Material

Oral_heart_axis_supplementary material.xlsx Oral_heart_axis_supplementary material.xlsx
